# IUC26682-89 Efficacy and tolerability of immune checkpoint inhibitors in older versus younger patients with metastatic renal cell carcinoma: a real-world Asian cohort

**DOI:** 10.1093/oncolo/oyag286.024

**Published:** 2026-08-21

**Authors:** R Kanesvaran, H S Tan, J W K Chan, W C Tan, D J M Ang, J J X Tan, H X Tan, A Roy Chowdhury

**Affiliations:** Division of Medical Oncology, National Cancer Centre Singapore - Singapore; Division of Medical Oncology, National Cancer Centre Singapore - Singapore; Division of Medical Oncology, National Cancer Centre Singapore - Singapore; Division of Medical Oncology, National Cancer Centre Singapore - Singapore; Division of Medical Oncology, National Cancer Centre Singapore - Singapore; Division of Medical Oncology, National Cancer Centre Singapore - Singapore; Division of Medical Oncology, National Cancer Centre Singapore - Singapore; Department of Geriatric Medicine, Singapore General Hospital - Singapore

## Abstract

**Background:**

Older adults are underrepresented in pivotal immune checkpoint inhibitor (ICI) trials for metastatic renal cell carcinoma (mRCC), resulting in uncertainty regarding treatment effectiveness and tolerability in routine clinical practice. We compared clinical outcomes and treatment-related toxicities between younger and older patients with mRCC receiving ICIs in a real-world Asian cohort.

**Methods:**

We conducted a retrospective study of 171 patients with mRCC treated with ICIs at the National Cancer Centre Singapore. Patients were stratified into younger (<65 years; n  =  96) and older (≥65 years; n  =  75) groups. Baseline demographic and clinicopathological characteristics, best objective response, treatment-related adverse events, progression-free survival (PFS), and overall survival (OS) were evaluated. Kaplan-Meier analysis with log-rank testing and Cox proportional hazards models were used for survival analyses.

**Results:**

Baseline characteristics were well balanced between age groups, with no significant differences in gender, race, histological subtype, ECOG performance status, prior nephrectomy or treatment line. The objective response rate was similar in younger and older patients (41.1% vs 37.0%, p  =  0.947). Grade 1–2 treatment-related toxicity occurred in 83% and 85% of younger and older patients, respectively (p  =  0.834). Older patients experienced numerically higher rates of grade 3–5 toxicity (41% vs 27%, p  =  0.072) and were significantly more likely to discontinue treatment because of toxicity (28% vs 15%, p  =  0.036). Median PFS was 9.2 months (95% CI 6.1–15.8) in younger patients and 9.7 months (95% CI 3.2–22.8) in older patients (HR 0.94, 95% CI 0.66–1.32; p  =  0.714). Median OS was 29.5 months (95% CI 18.4–39.2) and 31.5 months (95% CI 21.1–42.3), respectively (HR 1.14, 95% CI 0.78–1.66; p  =  0.491).

**Conclusions:**

Older patients with mRCC achieved comparable objective response, progression-free survival and overall survival following ICI therapy despite a higher likelihood of treatment discontinuation due to toxicity. These findings support the use of ICIs in appropriately selected older adults while emphasising the importance of proactive toxicity monitoring and individualized supportive care to optimise treatment delivery.

